# Pleural effusions in children undergoing cardiac surgery

**DOI:** 10.4103/0974-2069.64368

**Published:** 2010

**Authors:** Sachin Talwar, Sandeep Agarwala, Chander Mohan Mittal, Shiv Kumar Choudhary, Balram Airan

**Affiliations:** Cardiothoracic Center and Department of Pediatric Surgery, All India Institute of Medical Sciences, New Delhi, India

**Keywords:** Chylothorax, open heart surgery, pleural effusion

## Abstract

Persistent pleural effusions are a source of significant morbidity and mortality following surgery in congenital heart disease. In this review, we discuss the etiology, pathophysiology, and management of this common complication.

## INTRODUCTION

Pleural effusions are a significant problem following cardiac surgical procedures in children with a reported incidence of more than 25%.[1] They cause significant morbidity following cardiac surgery in children and contribute to their increased stay in the intensive care unit (ICU) and hospital. In this review, an attempt is made to discuss the etiology, pathophysiology, and general management guidelines for persistent pleural effusions and chylothorax following cardiac surgical procedures in children.

## INCIDENCE

Most of the data on the incidence of persistent effusions is derived from studies which comprise both the adult and pediatric population. In a study by Labidi *et al*,[1] of 2892 adult patients (mean age 66 years) undergoing open-heart surgery, 192 (6.6%) had a clinically significant pleural effusion within the first 30 days of the operation. Bocsi *et al*. reported that out of 75 patients (18 years of age or less) undergoing open-heart surgery, 29 (38.6%) developed a significant pleural effusion.[2] O'Callaghan *et al*. demonstrated effusions in 11% of 83 pediatric patients after cardiac surgery.[3] In our own experience with 348 patients undergoing univentricular repair, significant pleural effusion was encountered in 27% of the patients.[4] However, in one of the largest studies, involving 1341 pediatric patients undergoing correction of congenital heart disease, 18 (1.3%) were seen to have developed postoperative chylothorax.[5]

Pleural effusions are particularly common following univentricular palliation (bidirectional cavopulmonary or total cavopulmonary connection) and many studies have addressed the factors predisposed to increasing pleural drainage after the Fontan procedure.[6,7] Lower preoperative oxygen saturation, presence of postoperative infection, smaller conduit size, and longer duration of cardiopulmonary bypass have been associated with persistent pleural effusions after the extracardiac Fontan procedure.[6] Low pulmonary vascular compliance has also been shown to be an important risk factor for prolonged pleural effusion drainage after the extracardiac Fontan procedure.[7] The absence of a fenestration may also be associated with an increased incidence of pleural effusions following the lateral tunnel Fontan.[4]

## PATHOPHYSIOLOGY

Pleural effusions occur between two membranes: the visceral (inner) layer of the pleura attached to the lungs, and the parietal (outer) layer attached to the chest wall. The 'pleural space' is normally nonexistent and is lubricated by a very small amount of pleural fluid (10 – 20 ml) that provides lubrication between the two layers of the pleura. Fluid (sera) continuously moves from the parietal pleura through the pleural space to be absorbed by the visceral pleura. The fluid is then drained into the lymphatic system. This fluid in the pleural space is minimized by a balance of Starling forces, oncotic pressure in the circulation and negative pressure in the lymphatics of the lungs. Movement of the pleural fluid across the pleural space may be about 5 – 10 litres/day, and derangements in this movement may significantly increase the volume of the pleural fluid.

Risk factors for development of pleural effusion are listed in Table 1. Pleural effusion may occur in patients with (a) Increased capillary permeability caused by inflammation or infection (b) Increased hydrostatic pressure due to congestive heart failure, increased right-sided pressures or venous hypertension as in superior vena cava (SVC) obstruction (c) Decreased oncotic pressure from hypoalbuminemia, and (d) Increase in the normal negative pressure to a more negative intrathoracic pressure, secondary to atelectasis.

**Table 1:** Risk factors for prolonged pleural effusions and chylothortax after cardiac surgery in children

Systemic venous hypertension
Borderline pulmonary artery size
Peripheral pulmonary artery stenosis
Significant tricuspid regurgitation
Wound infection causing inflammation
Low preoperative oxygen saturation and polycythemia
Prolonged mechanical ventilation
Alterations in hormones that regulate fluid and electrolyte balance
Trauma to the thoracic duct and lymphatics

Probably the most common scenarios associated with prolonged pleural effusion after open-heart surgery are following the repair of the Tetralogy of Fallot (TOF) and following univentricular repair.[8,9] Systemic venous hypertension is often reflected by high right atrial pressures following repair of TOF, which increases the mean capillary hydrostatic pressure, leading to accumulation of excessive fluid in the pleural space.[10] In addition many of these patients may have borderline pulmonary artery and peripheral pulmonary artery stenosis, which leads to elevated right atrial pressures.[8,10] The presence of residual tricuspid insufficiency, compounds the problem of right heart failure. In patients with TOF and other forms of cyanotic heart disease, the pulmonary vascular anatomy is often abnormal. The muscular pulmonary arteries are thin-walled with medial atrophy and the lumens of the pulmonary arteries, veins, and capillaries are dilated. Once the obstruction to the right ventricular outflow tract is relieved, there is a sudden increase in the pulmonary blood flow resulting in increased intravascular hydrostatic pressure and an increased gradient across the pleural membrane, leading to effusion.[11] In these patients with preoperative polycythemia, there is an acute hemodilution as a result of surgery, resulting in an increased pulmonary blood flow, which leads to effusion.[9] Prolonged mechanical ventilation is also associated with an increased incidence of pleural effusion, due to an increase in the intrathoracic pressure, decrease in the intrapleural pressure, and an increase in the systemic venous pressure, which decreases the lymphatic drainage of the pleural cavities, leading to effusion.[12] Low preoperative oxygen saturation is often a reflection of the poor condition of the patient or a severe degree of obstruction to the right ventricular outflow tract, both of which are correlated with a higher incidence of effusion.

As mentioned above, postoperative pleural effusions are also very common following univentricular palliation and lead to a prolonged hospital stay of these patients. Attempts to address this issue have led to the concept of creating a fenestration in the Fontan pathway. However, the results and views on the role of fenestration in reducing the incidence of pleural effusion are conflicting.[4-7,9] The advent of extracardiac Fontan operation has not reduced the incidence of effusion significantly following this operation. On the contrary, use of a smaller conduit size during this operation has been associated with a higher incidence of effusion.[7] Performing this operation without cardiopulmonary bypass has also not altered the frequency of this problem, thus reinforcing the belief that the state of systemic venous hypertension imposed by the Fontan state is an important factor leading to effusions. Protein losing enteropathy following the Fontan operation may further increase the tendency to effusions, by reducing the plasma colloidal oncotic pressure. In addition, the occurrence of diaphragmatic palsy in some of these patients may adversely affect the Fontan circuit and lead to a higher incidence of effusions. The mechanisms of the latter are discussed in the article on diaphragmatic palsy in the same issue of this journal.[13]

Chylothorax occurs due to a direct trauma to the thoracic duct and the lymphatic system. This usually occurs following a Blalock-Taussig shunt, ligation of patent ducts arteriosus, or following coarctation of aorta repair via a thoracotomy approach.

## CLINICAL FEATURES

Postoperative pleural effusions may present in a variety of ways. Many times, the patients are completely asymptomatic and pleural effusions are an incidental finding on chest radiographs. However, depending on the size of the effusions, there may be a dramatic onset of dyspnea, shortness of breath, fever, and chest pain. Larger effusions can even lead to hemodynamic compromise due to cardiac embarrassment. In chylothorax, chest pain and fever are uncommon because chyle is not irritating to the pleural surface. More serious sequelae of chylothorax are malnutrition, weakness, dehydration, metabolic acidosis, and a compromised immunologic status.

## DIAGNOSIS

The plain chest radiograph is the most commonly performed investigation and may reveal findings ranging from opacification of the entire hemithorax in large effusions or mere blunting of the costophrenic angle in small effusions. The latter may be more apparent in chest radiographs obtained in the upright posture. The lateral decubitus film can also be used to diagnose small effusions. Bedside ultrasonography is also useful to diagnose pleural collections and to differentiate between effusions and lung consolidation or collapse.[9] High-resolution contrast chest tomograph (HRCT) has been promoted as the gold standard for diagnosis of small pleural collections, but carries a high radiation exposure. We, however, consider it an unnecessary and expensive investigation when a simple chest radiograph is equally informative.

Pleural fluid aspiration (Thoracentesis) using a needle helps in diagnosing the type of effusion and its etiology, which in turn helps to plan the management of pleural effusions. The appearance of the fluid, cell count, and certain biochemical tests as mentioned in Table 2, aid in the diagnosis.

**Table 2:** Criteria of exudative pleural effusion[15]

Pleural fluid protein-serum protein ratio greater than 0.5
Pleural fluid-serum lactate dehydrogenase (LDH) ratio greater than 0.6
Pleural fluid LDH more than two-thirds of the upper limit of normal serum LDH
Cholesterol level greater than 45 mg/dl
Serum-pleural albumin gradient less than 1.2 g/dl
Pleural-serum bilirubin ratio greater than 0.6
Prolonged mechanical ventilation
Alterations in hormones that regulate fluid and electrolyte balance
Trauma to the thoracic duct and lymphatics

The pleural fluid aspirate is straw-colored in patients with congestive heart failure and tuberculosis. It is a transudate in heart failure, whereas, it is an exudate in tuberculosis. In hemothorax it is bloody, in parapneumonic effusions it is serosanguinous, and in chylothorax it is milky white in color. Exudative pleural effusion is characterized by any one of the criteria listed in Table 2.[15] The cell content of tubercular effusion is predominantly lymphocytes, in acute infective pleural effusion it is neutrophils, and in hemothorax the pleural fluid hematocrit is > 0.5 of the blood hematocrit. Characteristics of pleural effusion due to various causes are detailed herewith:

### Effusion in congestive cardiac failure or raised venous pressure

The effusion is a transudate and is serous or straw-colored. It occurs more often on the right side. The effusion occurs because increased amount of lung interstitial fluid exits into the pleural space and overwhelms the capacity of the lymphatics in the parietal pleura to drain the fluid. Diagnostic thoracentesis is performed if effusions are not bilateral, if patient is febrile or has pleuritic chest pain.

### Hepatic hydrothorax

This may occur as a late complication of the Fontan procedure leading to liver cirrhosis and ascites.[16] The predominant mechanism is the direct movement of the peritoneal fluid through small holes within the diaphragm into the pleural space. The effusion is a transudate and is usually right-sided and large enough to produce severe dyspnea.

### Protein losing enteropathy

Loss of protein via the gastrointestinal tract occurs in 3.7% of the patients following the Fontan operation and is clinically characterized by fatigue, peripheral edema, pleural and pericardial effusions, ascites, and chronic diarrhea[17] associated with hypoalbuminemia.

### Parapneumonic effusions

Parapneumonic effusions are associated with bacterial pneumonia or lung abscess. These are exudative effusions and may be blood stained. Pleural effusions more than 10 mm thick on lateral decubitus film require a diagnostic tap.[18]

### Post cardiac injury syndrome

This occurs days to weeks following open-heart surgery. The development of pericardial, pleural, and pulmonary parenchymal inflammation following cardiac injury may be suggested by, fever, dyspnea, pleuropericardial rub, rales, elevated erythrocyte sedimentation rate, leukocytosis, pleural effusion, and pulmonary parenchymal infiltrates.[19] Nonetheless, these common clinical manifestations and laboratory features of post cardiac injury syndrome (PCIS) are nonspecific. The finding of a bloody, exudative pleural effusion with a pH greater than 7.30 helps to confirm a clinical suspicion of PCIS, but these pleural fluid characteristics are not pathognomonic.[20] Thus, arriving at a presumptive diagnosis of PCIS currently requires both clinical pattern recognition and exclusion of other diagnoses that may arise in this setting, namely, pulmonary embolism, congestive heart failure, atelectasis, and pneumonia.

### Tubercular pleuritis

Although by strict definition tubercular (TB) pleural effusions are not postoperative in nature, we have included a short discussion on these because these effusions are not uncommon in the cardiac intensive care unit in our country, even prior to open-heart surgery. These are thought to be due to a hypersensitivity reaction to the tuberculosis protein in the pleural space. Patients with tubercular pleuritis present with fever, weight loss, dyspnea, and/or pleuritic chest pain. Pleural fluid is straw-colored and exudative, with lymphocytic predominance. The diagnosis is established by demonstrating high levels of TB markers[21] in pleural fluid (adenosine deaminase > 45IU / l, Gamma interferon > 140 pg/ml or positive polymerase chain reaction-PCR for tuberculosis DNA).

### Chylothorax

Chylothorax represents chyle in the pleural cavity. Chyle is a lymphatic fluid rich in fat, and its digestive products are absorbed by the intestinal epithelium. Pleural fluid triglyceride levels have been used for diagnosis of chylothorax. Pleural fluid triglyceride levels > 110 mg/dl, presence of chylomicrons, low cholesterol level, and elevated lymphocyte count are diagnostic of a chylothorax.[22] When the pleural fluid triglyceride level is > 110 mg/dl, there is < 1% chance of it not being chylous, and pleural fluid with a triglyceride value of < 50 mg/dl has no more than a 5% chance of being chylous.[22] When the triglyceride level is between 55 and 110 mg/dl, a lipoprotein analysis is indicated, to detect chylomicrons. Other criteria for chylothorax include a pleural fluid to serum triglyceride ratio > 1, and a pleural fluid to serum cholesterol ratio < 1.[22] Pseudochylothorax, also known as chyliform pleural effusion or cholesterol pleural effusion, is the term applied to pleural fluid that mimics the true chylous pleural effusion in appearance, but lacks the biochemical criteria for chylothorax. In contrast to true chylothorax, it is chronic, and may be present for months to years. Pseudochylothorax is more likely to result from long-standing pleural effusion. The fluid is yellow or opalescent green and may initially be mistaken for a chylous effusion, an empyema, or both.[23] High cholesterol levels are typical of a pseudochylous pleural effusion. Cholesterol levels are generally > 200 mg/dl and may even exceed 1000 mg/dl.[24] The fluid may demonstrate rhomboid-shaped cholesterol crystals on microscopy, which do not stain with Sudan III stain.[24]

### Management

Management centers around correction of the underlying cause and drainage of pleural fluid either by needle aspiration or insertion of an intercostal tube.

Pleural effusion secondary to heart failure is managed by diuretic therapy and other drugs used for heart failure. The various drugs and their dosages have been elegantly summarized in a recent review.[25] They include digoxin, furosemide, spironolactone, captopril, enalapril, losartan, metoprolol, and carvedilol.

Protein losing enteropathy following the Fontan procedure is managed by dietary modifications (high protein and high-medium chain triglycerides), afterload reducing agents, ionotropic agents, heparin, albumin infusions, octreotide, prednisone, creation of atrial fenestration, if required Fontan revision or conversion to a different form, and in refractory cases, cardiac transplantation.[17]

Parapneumonic effusions that are uncomplicated generally resolve with antibiotics. In case of complicated effusions tube thoracostomy is required along with intravenous antibiotics. In case of loculated effusions instillation of a thrombolytic agent (streptokinase or urokinase) through tube is helpful.[18] Tubercular effusions are managed by standard anti-tubercular therapy.

**Figure 1:**
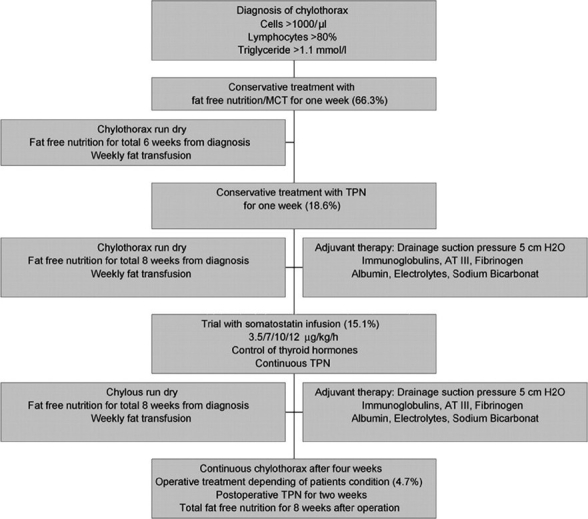
Treatment alogritham for chylothorax. [Reproduced with permission from: Cannizzaro V, Frey B, Bernet-Buettiker V. The role of somatostatin in the treatment of persistent chylothorax in children. Eur J Cardiothorac Surg. 2006;30:49-53.]

Various Options for the management of chylothorax are listed in Table 3 and a suggested management guideline is listed in the flow chart [Figure 1].

**Table 3:** Conservative therapies for managing chylothorax

Nil by mouth
Medium-chain triglycerides by mouth
Total parenteral nutrition
Drainage of chylothorax
Thoracentesis
Intercostal tube drainage
Ensuring complete lung expansion
Alterations in hormones that regulate fluid and electrolyte balance
Trauma to the thoracic duct and lymphatics

When managing chylothorax conservatively, chest tube drainage of less than 500 ml during the first 24 hours following cessation of oral intake and institution of total parentaral nutrition (TPN) may predict a successful outcome on conservative treatment.[26] Operative intervention for chylothorax should be considered when: (a) Average daily loss has exceeded 1500 ml/year of age in children for a five-day period,[27] (b) Chyle flow has not diminished over 14 days, (c) Nutritional depletion, fluid and electrolyte loss, hypolipemia, lymphocytopenia and immunodeficiency appear imminent, and (d) Accumulation of chyle is continuous and more than 5 ml/Kg per day despite chest tube drainage, after all conservative measures.

### Somatostatin or octreotide therapy

Somatostatin is an inhibitor of gastric, pancreatic, and intestinal secretions, and is known to decrease chyle production.[28] Octreotide, its analog, has been successfully used as a therapeutic adjunct for managing persistent chylothorax in a small number of neonatal patients and a larger number of pediatric patients.[28,29] No consensus has been reached as to the optimal route of administration, dose, duration of therapy, or strategy for discontinuation of therapy. The common doses are between 80 – 100 microg/kg/day and early initiation of this therapy has been recommended.[29]

### Pleurodesis

In the absence of a correctable cause, pleurodesis is recommended for persistent effusions / chylous drainage, unresponsive to medical and conservative measures. Almost all sclerosing agents[30] can produce fever, tachycardia, chest pain, and nausea. The patient needs to be premedicated with analgesics prior to instillation of the sclerosing agents. Talc is the most effective sclerosing agent used, and is often administered as a slurry.[31,32] When administered as a slurry through a chest tube or pleural catheter, it may be as effective as the direct insufflation of talc powder via thoracoscopy.[33] After drainage of the pleural fluid, the slurry (4 – 6 g of talc in a solution of 100 ml saline with or without lidocaine) is instilled. Adverse reactions include microemboli and granulomatous tissue reactions.[34] Tetracycline has been commonly used in the past in association with tube thoracostomy[35] and provides faster pleurodesis and pleural symphysis than chest tube drainage alone; however it is very painful. Doxycycline is an alternative to tetracycline and is equally effective.[36,37] Bleomycin (60 units) has been used as an alternative sclerosing agent and may have equivalent effectiveness to tetracycline. It is however, expensive and can have systemic toxicity.[38]

After placement of the chemical agent, the chest tube should be clamped for a period of two to four hours. During this time, the patient should lie in the following positions; supine, right lateral decubitus position, left lateral decubitus position, and for a period of time in the trendelenburg and reverse trendelenburg positions. Afterward, the chest tube should be unclamped and the residual fluid allowed to drain. This may be repeated a day or two later if high output continues.[39]

### Surgical therapy: Thoracic duct ligation

Lampson first demonstrated that chylothorax could be controlled by ligation of the thoracic duct.[40] Preoperative administration of lipophilic dye (e.g., Evans blue or cream) helps to locate the site of lymphatic leakage during the procedure. The thoracic duct is identified, isolated, and ligated just above the aortic hiatus between T-8 and T-12,[41] via a thoracotomy. Alternatively, an abdominal approach to ligate the thoracic duct is recommended, where a thoracic approach is not feasible.[42] Others have described a posterior extrapleural approach for ligation of the thoracic duct.[43] Even video-assisted thoracoscopic ligation is an option in bigger children and adults.[44]

### Pleuroperitoneal shunts

The pleuroperitoneal shunt has also been successfully used to manage persistent pleural drainage following open-heart surgery.[45] The shunt consists of pleural and peritoneal catheters connected to a manual pumping chamber capable of handling viscous fluid such as chyle. The system contains a one-way valve that allows drainage from the pleura into the peritoneal cavity where the fluid is absorbed. This prevents further loss of chyle and thus reduces the need for TPN.[46] Occlusion of the shunt occurs by fibrinous debris in 10% and needs replacement.[47]

Fluoroscopic percutaneous embolization of the thoracic duct

This is a minimally invasive alternative to open surgical ligation of the thoracic duct. Pedal lymphography is performed to opacify the lymphatic duct, for a puncture transabdominally.[48-50] Once access is obtained, a small catheter (3 – 4 Fr) is used and the contrast is injected to opacify the thoracic duct and collateral branches. Various coils and/or glue are used to occlude the thoracic duct. The success rate is variable, ranging from 45 to 100%.[49,50]

### Diaphragmatic fenestration

Another approach is to create a diaphragmatic fenestration for resistant chylothorax.[51,52] In our experience with two patients aged 5 and 12 years, we found that after thoracotomy, there was a diffuse ooze from the pleural surface rather than a localized leak, which meant that thoracic duct ligation in itself would be inadequate. In these patients, after breaking all loculi and adhesions, a 4 cm × 3 cm opening was made in the dome of diaphragm. An appropriately sized patch (polypropylene mesh in the first patient and a polytetrafluoroethylene patch with multiple 4 mm holes in the second patient) was chosen and sutured to the margins of the defect in the diaphragm. In both these patients, the chest tubes were removed on the seventh and fourth day, respectively. This procedure may therefore be a useful adjunct to thoracic duct ligation in managing these patients, when conventional modes of therapy have failed.

## SUMMARY

Persistent pleural effusions and chylothorax are multifactorial and are a significant cause of postoperative morbidity following open-heart surgery in children. It is important to diagnose them early and institute aggressive measures in the early postoperative period to achieve a satisfactory outcome. Management of persistent chylothorax can be particularly challenging, and at times, frustrating.
